# Tea production characteristics of tea growers (plantations and smallholdings) and livelihood dimensions of tea workers in Assam, India

**DOI:** 10.1016/j.dib.2018.02.056

**Published:** 2018-02-27

**Authors:** Eloise M. Biggs, Niladri Gupta, Sukanya D. Saikia, John M.A. Duncan

**Affiliations:** a UWA School of Agriculture and Environment, University of Western Australia, Perth, Australia; b Geography & Environment, University of Southampton, Southampton, UK; c Asian Disaster Preparedness Centre (RISU), Patna, India; d Tea Research Association, Tocklai Tea Research Institute, Jorhat, India; e College of Engineering and Informatics, National University of Ireland, Galway, Ireland, UK

## Abstract

This article provides summary data regarding tea production in Assam, India. Questionnaires were completed by tea producers and focus group discussions undertaken with tea workers. These data are presented for the four main tea growing regions of the state (Cachar, North Bank, South Bank and Upper Assam). Tables detail tea production characteristics of the tea plantations for both large- (> 10 ha) and small- (< 10 ha) holders. Figures provide supplementary information for research by Biggs et al. [1] regarding fertilizer application, landscape management strategies, healthcare provisioning and educational facilities within plantations, as well as detailing the livelihood dimensions of tea workers. The questions posed to producers are also included. For further context underpinning the research for which these data were collated, see ‘The tea landscape of Assam: multi-stakeholder insights into sustainable livelihoods under a changing climate’ by Biggs et al. [1].

**Specifications Table**

**Table:** 

Subject area	*Geography*
More specific subject area	*Human-environment interactions; livelihoods; climate change; sustainability; plantation agriculture*
Type of data	*Tables, figures, survey templates*
How data was acquired	*Focus group discussions and questionnaires*
Data format	*Summary*
Experimental factors	*Processed to summarize by tea growing region*
Experimental features	*This is a summary of processed raw data compiled by each of the main tea growing regions in Assam where data were collected*
Data source location	*Assam, India*
Data accessibility	*Provided with this article*

**Value of the data**

• Summary data provide comprehensive insights into tea production characteristics in the four main tea growing regions of Assam – these data could help target policy and investment more effectively to address environmental challenges such as climate change.
• Summary data provide comprehensive insights into the spatiality and socio-demographics of livelihoods for tea workers in the four main tea growing regions of Assam – these data could be used to help development agencies to identify where livelihood resilience strategies can be effectively supported to enhance social, human, physical, natural, and/or financial capital.
• Information harnessed from these data regarding the landscape of tea production in Assam can be used to identify cross-transferability of strategies from other tea-producing locations which are potentially suitable for adoption in Assam, to help build long-term social and environmental sustainability.
• These data can be used by international agencies seeking to increase sustainability and transparency in the tea value chain for India and the nation's trading partners.

## 1 Data

**Fig. 1:**
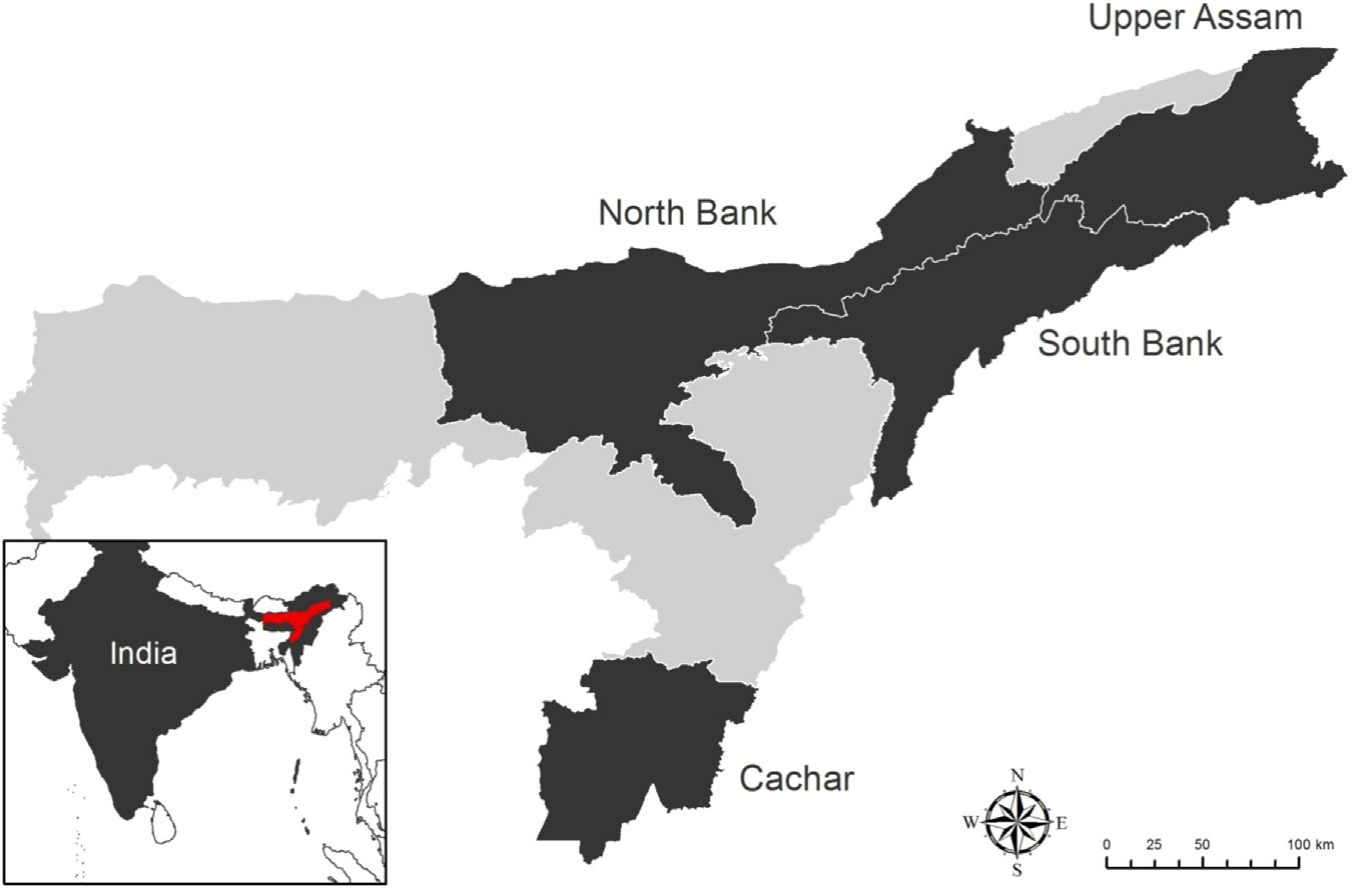
Location of the four main tea growing regions (Cachar, North Bank, South Bank and Upper Assam) in Assam, India; inset map indicates the state location [red] within the country.

This article provides summarized tables and figures regarding the role tea plays in sustaining the livelihoods of tea producers and workers in the four main tea growing regions (Cachar, North Bank, South Bank and Upper Assam) in the state of Assam, India (Fig. 1). In addition, the full questionnaires used for data collation from producers are provided for reference (see Survey 1 and Survey 2). Due to ethical compliance to retain anonymity, raw data are not provided, but summary data provided in this paper supplement and compliment that provided in Biggs et al. [1]. All data were collected between November 2014 and January 2016.

**Fig. 2:**
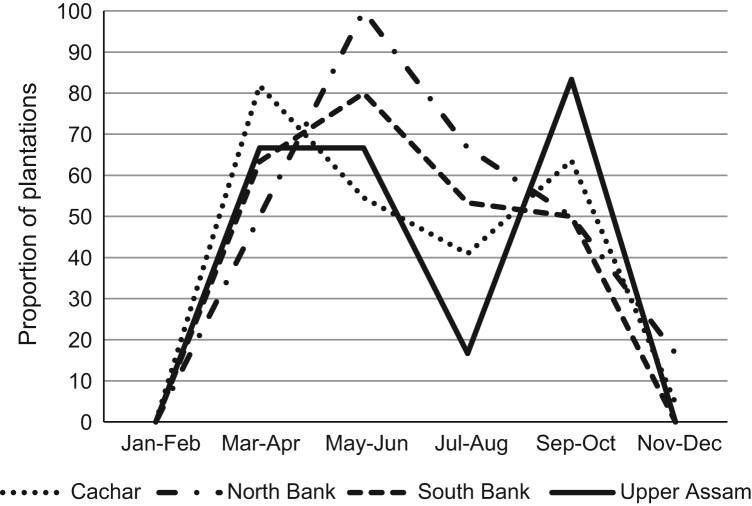
Time of year fertiliser is applied within each region as a proportion of total plantations.

Production characteristics for plantation estates are provided in Table 1. This summarizes information by tea growing region detailing estate attributes (time established, land under crop production, type and maturity of crops and yield), labor (number of workers, male-female split and technical staff), power supply and any green technology, certification status, other onsite agricultural activities, and where tea is sold (countries of sale). Application of inorganic fertilizer is also indicated (Table 1), with Fig. 2 providing information as to the months fertilizer is applied throughout the year. Production characteristics for smallholders are provided in Table 2. This summarizes information by tea growing region detailing estate attributes (time established and land under crop production), labor (number of workers, including those within the family of the producer), change in crop production over time, those who irrigate crops and apply inorganic fertilizer, membership of cooperatives, and certification status.

**Table 1:** Tea production characteristics of plantations included within research by Biggs et al. [1]. Where numerical ratio values are provided as responses, average values are provided for the region, with the range (minimum and maximum) indicated in brackets.

	**Cachar**	**North Bank**	**South Bank**	**Upper Assam**
Average time since tea established (years) (range)	114 (5-165)	123 (75-176)	113 (54-189)	104 (90-130)
Average land area owned under tea production (Ha) (range)	510 (208-1060)	493 (125-1141)	522 (125-992)	592 (432-730)
Average proportion of land owned/leased under tea production (%) (range)	80 (3-100)	86 (19-100)	96 (70-100)	98 (97-100)
Average area ratio of mature to young tea (range)	22 (0.2-99)	27 (15-51)	12 (0.7-36)	17 (11-30)
Modal yield (Ha)	<1500	1500-2000	1500-2000	1500-2000
Proportion growing Assam (A), Camod (Ca) and/or China (Ch) varieties of tea (%)	A 100	A 100	A 100	A 100
	Ca 91	Ca 66	Ca 42	Ca 17
	Ch 13	Ch 50	Ch 15	Ch 17
Number of workers (range)	1111 (400-3600)	1255 (543-2100)	1330 (161-5100)	1598 (1049-2084)
Average male-female worker ratio (range)	0.8 (0.4-1.25)	0.9 (0.4-1.6)	0.8 (0.1-1.4)	0.9 (0.7-1.1)
Average number of technical staff (range)	20 (3-95)	32 (3-55)	21 (5-62)	36 (16-62)
Predominant power supply	Coal-based with electricity	Coal-based	Coal- or gas-based with electricity	Gas-based with electricity
Proportion with green technology onsite (%)	0	17	18	0
Proportion of growers who apply inorganic fertiliser (%)	97	96	100	97
Proportion of growers with certification (%)	Organic 4	Organic 16	Organic 3	Organic 0
	Fairtrade 0	Fairtrade 0	Fairtrade 6	Fairtrade 0
	ISO 39	ISO 67	ISO 67	ISO 100
Other agricultural activities	Fisheries, rubber, spices, orchards	Spices, orchards (lemon), fisheries	Fisheries, orchards, spices, black pepper	Spices, orchards, black pepper, forestry
Countries of sale	Mainly India; Russia	Middle East (Iran, UAE); Russia; Europe (UK, Germany); USA; India	Mainly India; Middle East (Iran); Pakistan; Korea; Europe (UK, Germany); USA; Russia; China	Europe (UK, Germany); USA; Middle East (Iran)

**Table 2:** Tea production characteristics of smallholdings included within research by Biggs et al. [1]. ↑ indicates an increase, ↓ a decrease, and - no change over the time period smallerholders have been producing tea. Where numerical ratio values are provided as responses, average values are provided for the region, with the range (minimum and maximum) indicated in brackets.

	**Cachar**	**North Bank**	**South Bank**	**Upper Assam**
Average time since tea established (years) (range)	7 (1-28)	8 (3-20)	18 (13-25)	9 (1-20)
Average land area owned under tea production (ha) (range)	3.8 (2-9.5)	1.5 (0.3-8)	2 (0.1-9.4)	1.2 (0.3-4.4)
Average proportion of land owned under tea production (%) (range)	76 (0-100)	60 (0-100)	75 (0-100)	59 (0-100)
Number of workers (range)	9 (4-30)	6 (2-26)	8 (2-35)	5 (2-14)
Number of workers who are family members (range)	2 (1-5)	2 (0-8)	2 (0-7)	3 (1-8)
Proportion of growers with change in land area under tea cultivation (%)	0 ↓	2 ↓	0 ↓	7 ↓
	41 -	50 -	65 -	60 -
	59 ↑	48 ↑	35 ↑	33 ↑
Proportion of growers with change in yield of tea outputs (%)	6 ↓	6 ↓	37.5 ↓	16 ↓
	20 -	24 -	25 -	44 -
	74 ↑	70 ↑	37.5 ↑	40 ↑
Proportion of growers who irrigate crops (%)	38	52	0	3
Proportion of growers who are part of a cooperative (%)	39	69	0	9
Proportion of growers who apply inorganic fertiliser (%)	97	96	100	97
Number of growers with any certification	0	0	1 ISO	4 Organic

**Fig. 3:**
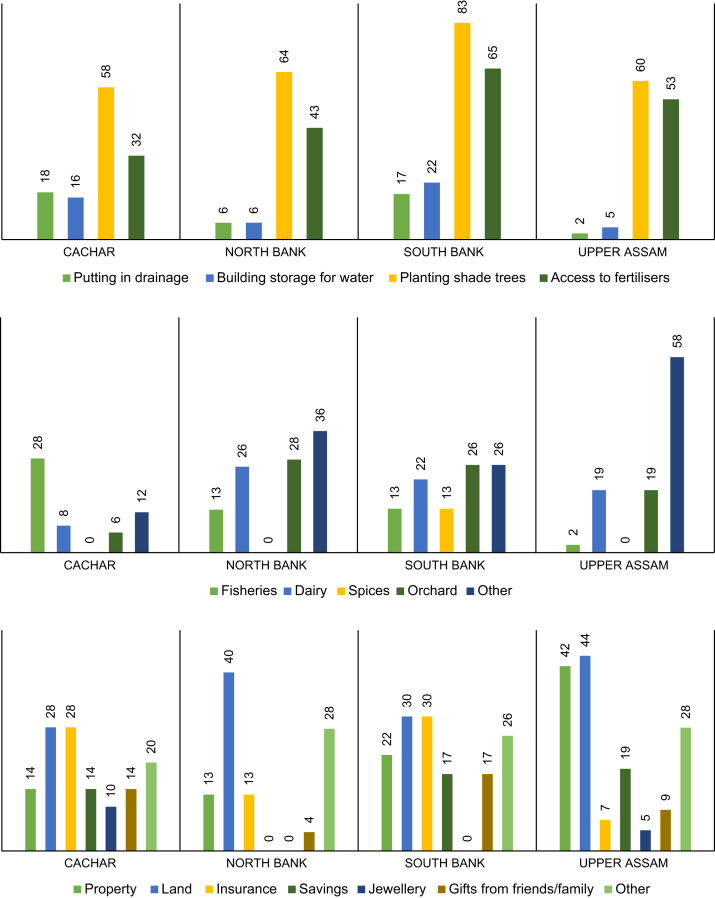
% proportion of smallholders with management strategies to help with: yield production (top), alternative income activities (middle), and additional assets should tea crop production fail (bottom.

**Fig. 4:**
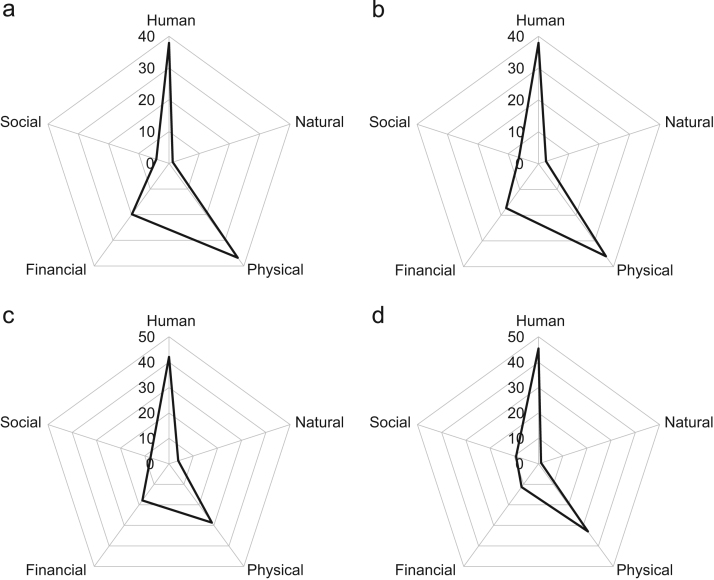
All assets identified by tea workers as important for sustaining livelihoods grouped by region for (a) Cachar (b) North Bank (c) South Bank and (d) Upper Assam.

**Fig. 5:**
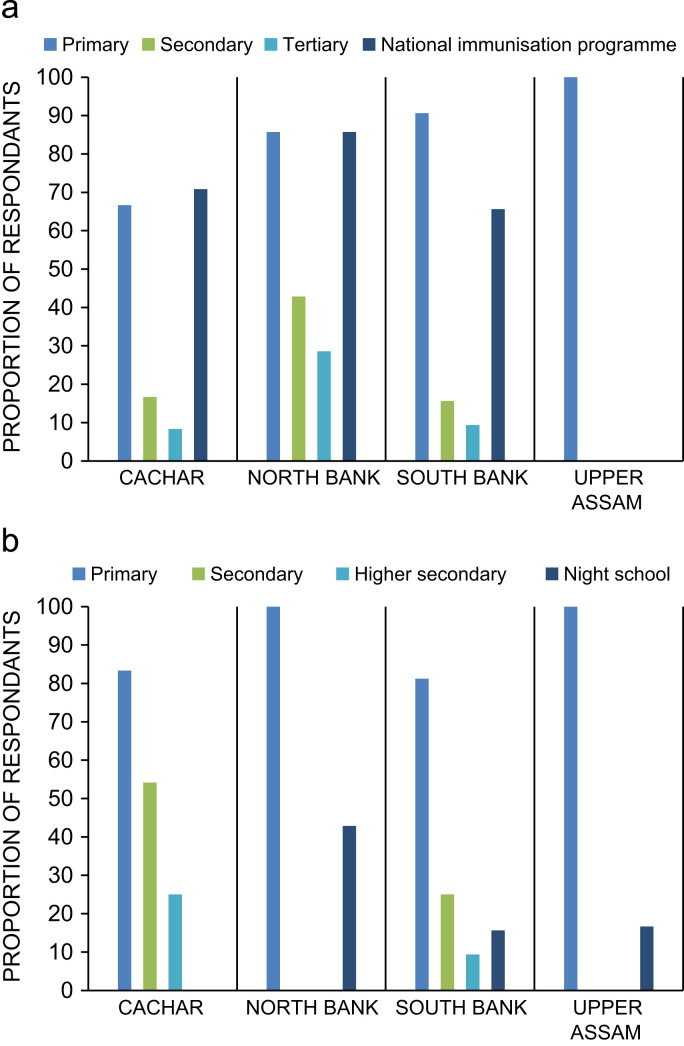
Health provisioning as proportion of total number of workshop respondents by region (top) and education provisioning as proportion of total number of workshop respondents by region (bottom).

The management strategies of smallholders to help address issues of shortfalls in field production, income and crop failure are indicated in Fig. 3. The crop failure component lists the assets which smallholders have access to should such a failure occur. Fig. 4 provides radar plots for each of the main tea growing regions indicating total human-natural-social-financial-physical assets stated by tea workers as important factors in sustaining their livelihoods within the tea plantations. Further details on the breakdown of assets by importance and gender are provided in Biggs et al. [1]. Fig. 5 provides details on plantation estates offering different types of health and/or education provisioning for tea workers.

## 2 Experimental design, materials and methods

Questionnaires: These were used to acquire the summary data provided in this article from tea producers. One questionnaire was used for plantation managers (see Survey 1) and another used for smallholders (see Survey 2). Following data collection, processing was undertaken to summarize findings into tables and figures which summarized the data by each of the tea growing regions. Questionnaire categories for fertilizer application were devised in relation to the Tea Research Association industry standards [3] to enable analysis of data as to where standards were being followed. In total, 48 plantation managers and 163 smallholders (50 Cachar; 47 North Bank; 23 South Bank; 43 Upper Assam) completed questionnaires for use in this research. Participants were identified via response to invitations sent to Tea Research Association member gardens to attend one of the workshops where questionnaires were undertaken (Table 3). Questionnaire data were entered by the main project researcher at Tocklai. Entries were checked for input errors and outliers by two other researchers. Where outliers were likely a respondent error by the participant (i.e. they misinterpreted the question units, or their response was illegible) these particular question answers were excluded from the analysis. Participants volunteered their time to participate in the workshops, and were provided with light food and drink refreshments.

**Table 3:** Number of plantation manager and smallholder participants for workshop attendance.

Location	Region(s)	Type	Date	Participants
Tocklai Tea Research Institute, Jorhat	North Bank, South Bank, Upper Assam	Plantation managers	7/1/2015	48
TRA Cachar Advisory Centre, Silcoorie	Cachar	Plantation managers	13/1/2016	23
Rangachakua, Sontipur	North Bank	Smallholders	12/11/2014	47
Tocklai Tea Research Institute, Jorhat	South Bank	Smallholders	18/11/2014	23
TRA Advisory Centre, Silcoorie	Cachar	Smallholders	3/12/2014	50
TRA Advisory Centre, Dikom	Upper Assam	Smallholders	12/12/2014	43

Focus group discussions: Focus group sessions were used to acquire the summary data provided in this article from tea workers. An adapted version of the Delphi technique was utilized to formulate group consensus regarding the role that tea has in sustaining tea workers’ livelihoods. The question ‘What are the important factors of working in a tea plantation which impact life for you and your family?’ was asked to respondents. All responses were noted by the session moderator. Following collection, those factors which could be classified as a human, physical, natural, financial or social asset were categorized following consultation to DFID's [2] sustainable livelihoods guidance sheets and discussion with a livelihoods expert independent to the project research. A total of 36 focus group discussions were held (18 with females; 18 with males) across 18 tea gardens (5 Cachar; 5 North Bank; 5 South Bank; 3 Upper Assam). The session moderators were trained in undertaking focus group discussions by researchers who have undertaken multiple focus group sessions on previous research projects. A pilot focus group session was run at the Tocklai Tea Research Institute tea estate with tea worker employees. Guidance on the concepts of the Delphi technique were also provided to the field researchers (focus group session moderators) prior to undertaking the focus groups. A detailed training session was provided which covered topics such as the need to remain neutral and impartial to the process in order not to bias the discussion of participants, ensure all participants opinions could be raised, and provide consistency in terms of how the sessions were administered across gardens. One of the moderators was fluent (native language) in Assamese (they led on the North Bank, South Bank and Upper Assam sessions), the other in Bengali (they led on the Cachar sessions), and both in English. Participants volunteered their time to participate in the sessions, and were provided with light food and drink refreshments.
