# Supplementary material for: Tea production characteristics of tea growers (plantations and smallholdings) and livelihood dimensions of tea workers in Assam, India
Source: Data Brief. 2018 Feb 27;17:1379–87. doi: 10.1016/j.dib.2018.02.056 (PMC5854876; doi:10.1016/j.dib.2018.02.056)
Supplement: Supplementary file 2 — Supplementary material [file mmc2.doc]

| **Assessing the role of tea in the landscapes of Assam**  Plantation Tea Estates  Dr Eloise Biggs (University of Southampton) and Dr Niladri Gupta (Tea Research Association) Ethics reference: 11950 | | | |  |
| --- | --- | --- | --- | --- |
| *Please answer all of the following questions.* |  |  |  | |

# Information on your tea estate 1. Which District is your tea estate in? 2. Which Block is your tea estate in? 3. What is the name of your company (not the name of the tea estate)? 4. What year was your tea estate established? 5. What is the area of your tea garden? 6. What is the area of Young Tea? 7. What is the area of Mature Tea? 8. What is the status of the tea estate land? (tick one) ⃝ Owned ⃝ Leased 9. How many tea labourers do you employ? 10. How many are male? 11. How many are female? 12. How many technical staff do you employ on your estate? 13. Do you use any green technology in your tea garden? ⃝ Solar ⃝ Wind ⃝ Biogas ⃝ Other (please specify) 14. What powers your tea factory? ⃝ Coal-based ⃝ Gas-based ⃝ Other (please specify) 15. Does your tea garden have any of the following certifications? ⃝ Organic ⃝ Fairtrade ⃝ ISO 16. Which countries are buyers of your tea? Please list in order of highest to lowest buyers by revenue.

# 17. Do you have any meteorological observatory in your estate? ⃝ Yes ⃝ No 18. If yes, please specific all of the parameters you record: Information on your tea production 19. What variety of tea clone do you grow in your garden? ⃝ Assam variety ⃝ Cambod variety ⃝ China variety 20. How often are the tea leaves plucked? 21. What is the average yield of your garden? ⃝ < 1500 ⃝ 1500 - 2000 ⃝ 2000 - 2500 ⃝ 2500 - 3000 ⃝ > 3000 kg/ha 22. Do you engage with any other activities besides tea production on your estate ⃝ Fisheries ⃝ Dairy ⃝ Spices ⃝ Orchards ⃝ Other (please specify) 23. Where do you get your water supply from for your tea crops? (tick those which apply) ⃝ Rainfall ⃝ Bore hole (groundwater) ⃝ Ponds ⃝ River ⃝ Other (please specify) 24. Do you irrigate your tea crops? 25. If yes, what type of irrigation? ⃝ Yes ⃝ No ⃝ Drip irrigation ⃝ Sprinkler 26. If yes, how often do you irrigate? 27. Do you carry out soil rehabilitation before new planting? ⃝ Yes ⃝ No 28. Do you use any of the following soil conservation practices? (tick those which apply) ⃝ Low tillage ⃝ Contour Planting ⃝ Afforestation ⃝ Controlled and Strip Weed ⃝ Infilling vacancies ⃝ Proper Shade ⃝ Contour Drains ⃝ Grassy Waterways ⃝ Mulching ⃝ Permanent grass strip along contour drains ⃝ Other (please specify) 29. Do you use any of the following water conservation practices? (tick those which apply) ⃝ Check Dam ⃝ Ponds ⃝ Other (please specify) 30. Do you use any of the following natural pest control practices? (tick those which apply) ⃝ Light Trapping ⃝ Physical collection after killing them ⃝ Chrysalid Collection ⃝ Delta tap with sticky liner ⃝ Bacterial spray for termites ⃝ Bacterial spray for tea mosquito bug ⃝ Botanical formulations ⃝ Other (please specify) 31. Do you use any of the following organic fertilisation on your tea garden? (tick those which apply) ⃝ None ⃝ Peat ⃝ Animal Waste ⃝ Plant Waste ⃝ Sewage ⃝ Other (please specify) 32. Do you use inorganic fertilisers on your tea garden? ⃝ Yes ⃝ No (if no, skip questions 33-45) 33. What time of year do you apply fertilisers? ⃝ N/A ⃝ Jan – Feb ⃝ Mar – Apr ⃝ May – Jun ⃝ Jul – Aug ⃝ Sep – Oct ⃝ Nov - Dec 34. Please describe the methods you use for fertiliser application: 35. How much Nitrogen (N) do you apply [kg/ha]? ⃝ < 90 ⃝ 90 - 110 ⃝ 110 - 140 ⃝ 140 – 165 ⃝ > 165 36. How much Phosphorus (P_2_O_5_) do you apply [kg/ha]? ⃝ < 20 ⃝ 20 - 30 ⃝ 30 - 40 ⃝ 40 - 50 ⃝ > 50 37. How much Potassium (K_2_O) do you apply [kg/ha]? ⃝ < 50 ⃝ 50 – 70 ⃝ 70 - 80 ⃝ 80 - 90 ⃝ 90 - 100 ⃝ 100 – 110 ⃝ 110 – 120 ⃝ 120 – 140 ⃝ 140 – 165 ⃝ > 165 38. What is the YTD mixture ratio used for Young Tea in your garden? : : per 100kg mixture 39. What is the frequency of application for the YTD mixture? ⃝ 1 – 3 weeks ⃝ 4 – 6 weeks ⃝ 6 – 8 weeks ⃝ 9 – 11 weeks ⃝ > 12 weeks 40. How much Sulphur (S) do you apply [kg/ha]? ⃝ < 10 ⃝ 10 – 20 ⃝ 20 – 30 ⃝ 30 – 40 ⃝ 40 – 50 ⃝ > 50 41. How much Zinc Sulphate (ZnSO_4_) do you apply to your tea [kg/ha/year]? ⃝ None ⃝ < 10 ⃝ 10 - 11 ⃝ 11 – 12 ⃝ 12 – 13 ⃝ 13 – 14 ⃝ > 14 42. How much Magnesium Sulphate (MgSO₄) do you apply to your tea [% concentration]? ⃝ None ⃝ < 1 ⃝ 1 – 2 ⃝ 2 – 3 ⃝ 3 – 4 ⃝ 4 – 5 ⃝ > 5 43. How much Dolomite do you use to tackle low soil pH [Tonnes/Ha]? ⃝ None ⃝ < 1 ⃝ 1 – 2 ⃝ 2 – 3 ⃝ 3 – 4 ⃝ 4 – 5 ⃝ > 5 44. What time of year do you apply the Dolomite? ⃝ N/A ⃝ Jan – Feb ⃝ Mar – Apr ⃝ May – Jun ⃝ Jul – Aug ⃝ Sep – Oct ⃝ Nov - Dec 45. How do you manage issues of high soil pH? Information on your social programmes 46. What healthcare does your tea estate provide for your tea workers? ⃝ Primary ⃝ Secondary ⃝ Tertiary ⃝ National immunization programme 47. What education facilities do you have on your tea estate? ⃝ None ⃝ Primary ⃝ Secondary ⃝ Higher Secondary ⃝ Night School (adults) 48. If your tea garden provides social awareness programmes, please tell us what these are: 49. Does your tea estate have a worker’s grievance forum (lok adalat)? ⃝ Yes ⃝ No 50. Do staff from your tea estate attend Area Scientific Committee (ASC) meetings held at TRA? ⃝ Yes ⃝ No 51. How many of your technical staff do you send per year for field/management training? 52. Do you think weather is a threat to your tea production? (tick one) ⃝ Yes ⃝ No ⃝ Possibly 53. Briefly provide your thoughts on why/how you think weather is/is not impacting your tea production: Please now take a short break to listen to our presentation on our research and information on regional climate issues impacting Assam.

# 54. Do you think climate change is at all an issue for you and your plantation? ⃝ Definitely Yes ⃝ Yes ⃝ Maybe ⃝ No ⃝ Definitely No Please provide a reason(s) here as to why you selected the above answer: 55. Were you aware of climate change issues from any other sources before coming to this workshop? ⃝ Yes ⃝ No If yes, please specific what: 56. In your opinion, what do you think are best solutions for dealing with changing climate in Assam?

# 57. Do you think intercropping or other activities in your tea garden would enable you to increase land productivity and compensate for any loss in tea yield due to changing climate? ⃝ Yes ⃝ No If yes, please specific what activities you would consider undertaking:

# 58. How likely are you to follow the TRA advised management practices in view of changing climate? ⃝ Very likely ⃝ Likely ⃝ Not sure ⃝ Unlikely ⃝ Very unlikely Please provide any further comments here:

*59. How likely are you to take up irrigation if a subsidy is provided?***⃝ Very likely ⃝ Likely ⃝ Not sure ⃝ Unlikely ⃝ Very unlikely**
*60. Do you feel the information you received today was valuable and that the workshop was worthwhile for you to attend?*Please tell us your views:
 *Thank you very much for taking the time to complete this survey and attend our workshop. The information you have provided will be very valuable for our research.*
