# Supplementary material for: Tea production characteristics of tea growers (plantations and smallholdings) and livelihood dimensions of tea workers in Assam, India
Source: Data Brief. 2018 Feb 27;17:1379–87. doi: 10.1016/j.dib.2018.02.056 (PMC5854876; doi:10.1016/j.dib.2018.02.056)
Supplement: Supplementary file 3 — Supplementary material [file mmc3.doc]

| **Assessing the role of tea in the landscapes of Assam**  Smallholder Tea Estates  Dr Eloise Biggs (University of Southampton) and Dr Niladri Gupta (Tea Research Association) Ethics reference: 11950 | | | |  |
| --- | --- | --- | --- | --- |
| *Please answer all of the following questions* |  |  |  | |

# Information on your tea estate 1. Which District is your tea estate in? 2. Which Block is your tea estate in? 3. How much land area do you own? 4. How much area of this land is used for producing tea? 5. How many people work in your tea garden? 6. How many are male? 7. How many are female? 8. How many are members of your family?

# Information on your income 9. What proportion of your annual income comes from growing tea? (tick one) ⃝ 0 – 19 % ⃝ 20 – 39 % ⃝ 40 – 59 % ⃝ 60 – 79 % ⃝ 80 – 99 % ⃝ 100 %

**10. If this proportion is less than 100%, what other sources of income-generating activities do you engage in? (tick all that apply)**
***⃝*** Agricultural crops ***⃝*** Daily labour ***⃝*** Fishing ***⃝*** Receiving remittances
***⃝*** Other (please specify)


**Information on your tea production
11. How many years have you been growing tea on your land?**

**12. Has the amount of land you have used for producing tea changed? (tick one)**
***⃝*** Stayed the same ***⃝*** Increased ***⃝*** Decreased
**13. Has the yield from tea changed over the time you have produced tea on your land? (tick one)**
***⃝*** Stayed the same ***⃝*** Increased ***⃝*** Decreased

**14. How often do you pluck the tea leaves?**

**15. Where do you get your water supply from for your tea crops? (tick those which apply)**
***⃝*** Rainfall ***⃝*** Bore hole (groundwater) ***⃝*** Ponds ***⃝*** River ***⃝*** Other (please specify)

**16. Do you irrigate your tea crops? 17. If yes, what type of irrigation?**
***⃝*** Yes ***⃝*** No ***⃝*** Drip irrigation ***⃝*** Sprinkler
**18. If yes, how often do you irrigate?**

**19. Do you have the capacity to do any of the following? (tick those which apply)**
*⃝* Build storage for water *⃝* Plant shade trees *⃝* Put in drainage *⃝* Access fertilisers
**20. Does your tea garden have any of the following certifications?**
***⃝*** Organic ***⃝*** Fairtrade ***⃝*** ISO
**21. Does your tea garden belong to cooperative?**
***⃝*** Yes ***⃝*** No
**22. Do you engage with any other activities besides tea production on your estate**
***⃝*** Fisheries ***⃝*** Dairy ***⃝*** Spices ***⃝*** Orchards ***⃝*** Other (please specify)

**23. Do you use *inorganic* fertilisers on your tea garden?**
***⃝*** Yes ***⃝*** No (if no, skip questions 25-27)
**24. What time of year do you apply fertilisers?**
***⃝*** N/A  ***⃝*** Jan – Feb ***⃝*** Mar – Apr ***⃝*** May – Jun ***⃝*** Jul – Aug ***⃝*** Sep – Oct ***⃝*** Nov - Dec
**25. Please describe the methods you use for fertiliser application:**


**Information on your livelihoods
26. If your tea crops fail, do you have other assets you can draw upon? (tick those which apply)**
***⃝*** Property ***⃝*** Land ***⃝*** Insurance ***⃝*** Savings ***⃝*** Jewellery ***⃝*** Gifts from family/friends
***⃝*** Other (please specify)

**27. Drawing upon these assets, how many tea crop failures do you think you could sustain?**
***⃝*** 1 ***⃝*** 2 – 3 ***⃝*** 4 – 5 ***⃝*** 6+
**28. If you no longer received subsidies for tea production would you still grow tea? (tick one)**
***⃝*** Yes ***⃝*** No ***⃝*** Possibly
**29. If no or possibly, what would you grow on the land you are currently growing tea on?**

**30. How many workshops organised by the Tea Board of India and/or Tocklai Tea Research Institute have you attended since you started growing tea?**

**31. Do you think weather is a threat to your tea production? (tick one)**
***⃝*** Yes ***⃝*** No ***⃝*** Possibly
**32. Briefly provide your thoughts on why/how you think weather is/is not impacting your tea production:**


**Please now take a short break to listen to our presentation on our research and information on regional climate issues impacting Assam.**

**33. Do you think climate change is at all an issue for you and your plantation?**⃝ Definitely Yes ⃝ Yes ⃝ Maybe ⃝ No ⃝ Definitely No
Please provide a reason(s) here as to why you selected the above answer:
 **34. Were you aware of climate change issues from any other sources before coming to this workshop?**⃝ Yes ⃝ No
If yes, please specific what:
 **35. In your opinion, what do you think are best solutions for dealing with changing climate in Assam?**

**36. Do you think intercropping or other activities in your tea garden would enable you to increase land productivity and compensate for any loss in tea yield due to changing climate?**⃝ Yes ⃝ No
If yes, please specific what activities you would consider undertaking:

**37. How likely are you to follow the TRA advised management practices in view of changing climate?**⃝ Very likely ⃝ Likely ⃝ Not sure ⃝ Unlikely ⃝ Very unlikely
Please provide any further comments here:

**38. How likely are you to take up irrigation if a subsidy is provided?**⃝ Very likely ⃝ Likely ⃝ Not sure ⃝ Unlikely ⃝ Very unlikely

**39. Do you feel the information you received today was valuable and that the workshop was worthwhile for you to attend?**Please tell us your views:
 *Thank you very much for taking the time to complete this survey and attend our workshop. The information you have provided will be very valuable for our research.*
